# Case Report: Gitelman syndrome with a suspected MEFV- associated autoinflammatory phenotype: diagnostic challenges in a complex case

**DOI:** 10.3389/fimmu.2026.1784807

**Published:** 2026-03-25

**Authors:** Yu Li, Yun Lu, Yue Wang, Xiaotian Yang, Qiuchi Zhang, Yu Xie

**Affiliations:** 1 Nanjing University of Chinese Medicine, Nanjing, Jiangsu, China; 2 Department of Neurology, First Affiliated Hospital, Nanjing University of Chinese Medicine, Nanjing, Jiangsu, China; 3 Department of Rheumatology and Immunology, First Affiliated Hospital, Nanjing University of Chinese Medicine, Nanjing, Jiangsu, China

**Keywords:** autoinflammatory phenotype, diagnostic challenges, genetic diagnosis act, Gitelman syndrome (GS), MEFV variant

## Abstract

We report a rare case of genetically confirmed Gitelman syndrome (GS) in a Chinese female patient presenting with systemic inflammatory manifestations and a heterozygous MEFV variant of uncertain significance. The patient initially exhibited arthritis, elevated inflammatory markers, and a positive labial gland biopsy, raising suspicion for Sjögren’s syndrome. However, absence of sicca symptoms and failure to meet the 2016 ACR/EULAR classification criteria excluded a definitive diagnosis. Persistent hypokalemia, hypomagnesemia, and metabolic alkalosis led to genetic testing, which identified compound heterozygous pathogenic variants in SLC12A3, confirming GS. Although the MEFV variant suggested a possible autoinflammatory phenotype, established diagnostic criteria for familial Mediterranean fever were not fulfilled. This case highlights the diagnostic complexity arising from overlapping renal and inflammatory features and underscores the importance of cautious genetic interpretation and multidisciplinary evaluation in complex multisystem presentations.

## 1 Introduction

Gitelman syndrome (GS) is an autosomal recessive renal tubular disorder caused by pathogenic variants in the SLC12A3 gene, which encodes the thiazide-sensitive sodium–chloride cotransporter (NCC) in the distal convoluted tubule. Dysfunction of NCC leads to impaired sodium and chloride reabsorption and results in characteristic biochemical abnormalities, including hypokalemia, hypomagnesemia, hypocalciuria, hypochloremic metabolic alkalosis, hyperreninemia, and secondary hyperaldosteronism, typically in the setting of normal or low blood pressure (1–4). GS is generally considered a primary inherited tubulopathy; however, its presentation may occasionally overlap with systemic inflammatory or autoimmune conditions, complicating diagnosis.

Familial Mediterranean fever (FMF; MIM: 134610 and 249100) is a hereditary autoinflammatory disorder caused by variants in the MEFV gene and characterized by recurrent episodes of fever and serositis. Although classically inherited in an autosomal recessive pattern, autosomal dominant forms with incomplete penetrance have been described (5).Diagnosis remains primarily clinical and is supported by genetic findings; however, interpretation of MEFV variants of uncertain significance may pose challenges in patients with atypical inflammatory manifestations.

Here, we report a Chinese female patient with genetically confirmed GS who presented with systemic inflammatory features and a heterozygous MEFV variant of uncertain significance. The coexistence of electrolyte disturbances and inflammatory manifestations initially led to diagnostic uncertainty, including consideration of autoimmune and autoinflammatory diseases. This case highlights the complexity of evaluating multisystem presentations and emphasizes the importance of integrating genetic data with careful clinical assessment.

## 2 Case presentation

In February 2025, a 52-year-old woman presented to Taizhou Jiangyan Chinese Medicine Hospital with a sudden onset of right hip pain and limited mobility following prolonged sitting and fatigue. A bilateral hip MRI without contrast revealed a left femoral neck herniation pit (Pitt’s pit), right hip synovial bursa effusion suggestive of bursitis, dilation of the right iliopsoas bursa, and mild edema of the surrounding soft tissues. A small amount of fluid was also noted in the left hip joint. She was hospitalized and treated with anti-inflammatory and rehydration therapy, edema reduction, analgesia, hepatoprotective agents, and potassium supplementation. During hospitalization, she developed a low-grade fever (37.5 °C). Her symptoms improved with treatment, and she was discharged.

Ten days prior to the current admission, the patient experienced worsening right hip pain after prolonged sitting at work, accompanied by pain in the lateral aspect of the right knee and right ankle, along with restricted movement. She presented to the Orthopedics Department of our hospital for further evaluation and was admitted with a diagnosis of “right hip synovitis.”

Upon admission, serial laboratory testing confirmed persistent hypokalemia. An extended immunological workup demonstrated negative antinuclear antibodies (ANA) and anti-extractable nuclear antigen (ENA) antibodies. The ENA panel specifically included anti-nRNP/Sm, anti-Sm, anti-U1RNP, anti-SSA/Ro, anti-SSB/La, anti-Scl-70, anti-Jo-1, anti-centromere, anti-nucleosome, anti-histone, anti-ribosomal P protein, anti-PM-Scl, anti-mitochondrial M2, anti-PCNA, and anti-Ro-52 antibodies, all of which were negative. Notable findings included elevated complement C3 (1.66 g/L), C4 (0.41 g/L), and C-reactive protein (87.90 mg/L). Rheumatoid factor was within normal limits (<20.0 U/mL). Serology was positive for IgG antibodies against cytomegalovirus (56.38 U/mL), rubella virus (11.95 IU/mL), Toxoplasma gondii (95.14 IU/mL), and herpes simplex virus type 1 (14.06 COI). Serum electrolyte evaluation demonstrated hypokalemia, hypomagnesemia, hypochloremia, and mild hyponatremia, accompanied by elevated serum bicarbonate, suggestive of metabolic alkalosis. Serum calcium and phosphorus levels were within normal ranges. Arterial blood gas analysis revealed a primary metabolic alkalosis characterized by alkalemia (pH 7.47), elevated bicarbonate concentration (28 mmol/L), and positive base excess (+4 mmol/L). This was accompanied by hypokalemia and hypochloremia, consistent with a renal tubular electrolyte disorder, as shown in **Tables 1**, **2**.

**Table 1:** Biological markers and complete blood count.

Parameter	Result	Reference range
Height	159cm	
Weight	58kg	
ANA	Negative	
ENA	Negative	
HLA-B27	Not detected	
Erythrocyte sedimentation Rate (ESR)	99mm/h	0–20 mm/h
CRP	87.9mg/L	<8mg/L
RF	<20U/ml	<20U/ml
Anti-streptolysin O(ASO)	34.00	<116.00 IU/ml
Complement 3	1.66g/L	
Complement 4	0.41g/L	
Serum electrolyte		
Serum potassium	2.63mmol/L	3.50-5.10mmol/L
Serum sodium	135.4 mmol/L	136–146 mmol/L
Serum chloride	92.7 mmol/L	97–108 mmol/L
Serum bicarbonate	30.2 mmol/L	22–29 mmol/L
Serum magnesium	0.65mmol/L	0.75-1.02 mmol/L
Serum calcium	2.55 mmol/L	2.10-2.90 mmol/L
Serum phosphorus	1.19 mmol/L	0.85-1.19mmol/L
Blood glucose	9.39 mmol/L	3.89-6.11 mmol/L
Fasting blood-glucose	7.32mmol/L	3.89-6.11mmol/L
Complete blood count		
White blood cell count	8.78×10^9/L	4.00-10.00×10^9/L
Hemoglobin	104 g/L	120–160 g/L
Platelet	322×10^9^/L	125-350×10^9^/L
24-hour urinary protein quantity	354mg/24 h	<150 mg/24 h
24-hour urine output	2.7L	1.0-2.0L
Aldosterone-to-Renin Ratio (ARR or AARR)	0.19	
Plasma Aldosterone (pg/mL) Angiotensin II (pg/mL)	224.97 63.88	40–310 49–252
Plasma Renin (pg/mL)	116.32	3.8–38.8
Adrenocorticotropic Hormone (ACTH) (pg/mL)	13.27pg/ml	7.20–63.3pg/ml

**Table 2:** Arterial blood gas and serum electrolyte analysis.

Parameter	Result	Reference range	Interpretation
pH (temperature corrected)	7.47	7.35–7.45	Elevated (alkalemia)
PaO_2_ (mmHg)	96	83–108	Normal
PaCO_2_ (mmHg)	37.8	32–48	Normal
Oxygen saturation (%)	98	95–99	Normal
Potassium (mmol/L)	3.1	3.4–4.5	Decreased
Sodium (mmol/L)	135	135–145	Lower limit of normal
Chloride (mmol/L)	94	98–106	Decreased
Ionized calcium (mmol/L)	1.17	1.15–1.29	Normal
Serum bicarbonate (HCO_3_⁻, mmol/L)	28	21–28	Upper limit of normal
Base excess (mmol/L)	+4	-3 to +3	Elevated
Lactate (mmol/L)	1.7	0.5–1.6	Slightly elevated
Glucose (mmol/L)	20.5	3.9–5.8	Elevated
Hemoglobin (g/dL)	10	12–17	Decreased
Anion gap (mmol/L)	12.6	8–16	Normal
p50 (mmHg)	24	25–29	Decreased

Given these findings, a rheumatologic/autoimmune disorder was strongly suspected. Consequently, the patient was transferred to the Rheumatology and Immunology Department of our hospital for further investigation and management.

**Figure 1:**
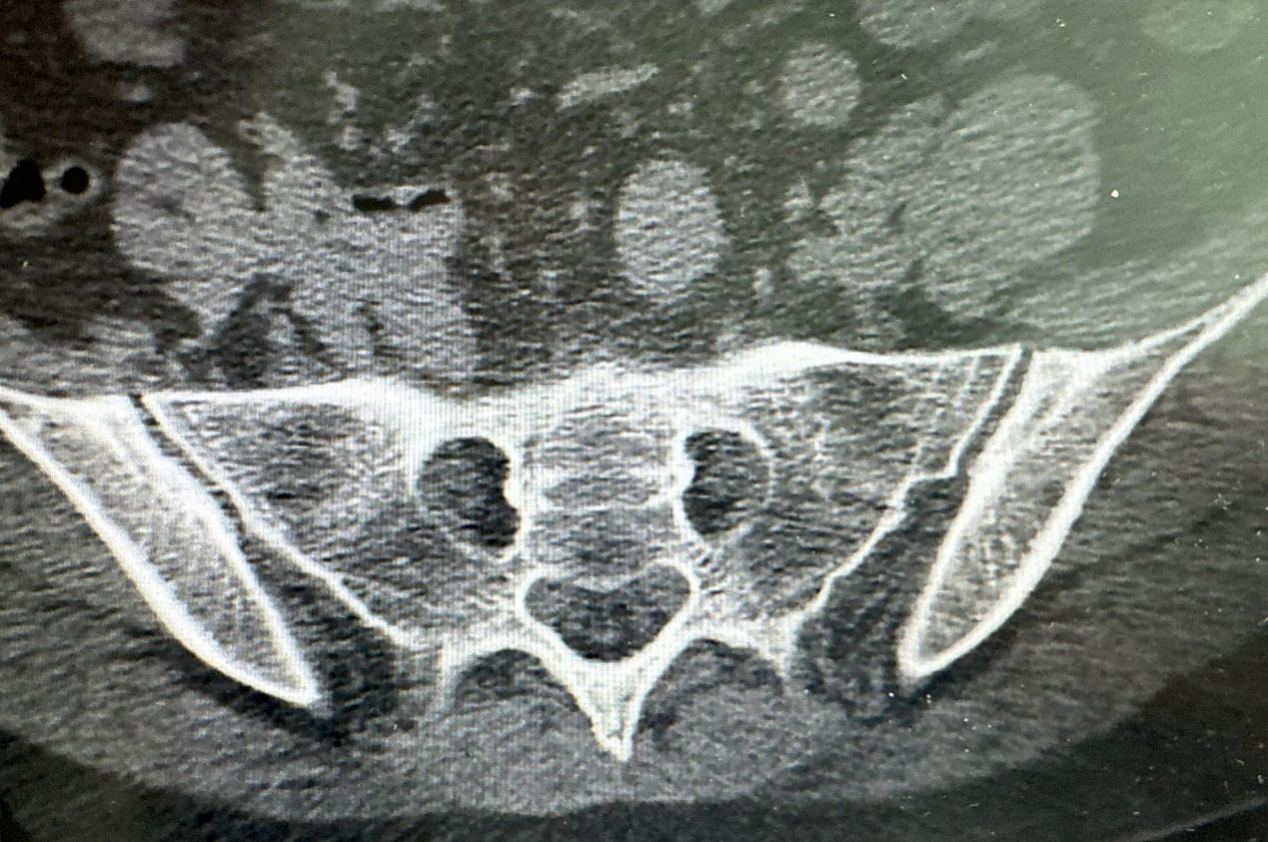
Joint CT shows findings suggest likely bilateral osteitis condensans ilii.

Bilateral sacroiliac joint CT shows Findings suggest likely bilateral osteitis condensans ilii. Hip joint plain MRI scan shows Minimal bone edema is noted in the right femoral head and neck region. A cystic lesion is observed in the left femoral neck, possibly representing a synovial herniation. Both hip joints show minimal effusion and synovitis. Labial gland excision specimen shows the interstitium shows three foci of lymphocyte and plasma cell infiltration per 4 mm², consistent with chronic inflammatory cell infiltration (Grade IV), as shown in **Figure 1**.

Sjögren’s syndrome was initially suspected based on the presence of focal lymphocytic infiltration on labial gland biopsy. However, comprehensive evaluation—including extended serological testing (anti-SSA/Ro, anti-SSB/La, anti-Ro-52 antibodies), hematologic investigations, serum electrolyte analysis, renin–angiotensin–aldosterone profiling, and arterial blood gas analysis—was subsequently performed to clarify the diagnosis. Despite the positive histopathological finding, the patient had never experienced sicca symptoms such as xerostomia or xerophthalmia. Serological testing for Sjögren’s-associated autoantibodies was negative. According to the 2016 ACR/EULAR classification criteria, the total score was 3 (based solely on labial gland histopathology), which does not meet the diagnostic threshold (≥4) for primary Sjögren’s syndrome. Therefore, a definitive diagnosis of Sjögren’s syndrome was not established.

Prior to genetic testing, the patient was treated empirically under the working diagnosis of autoimmune-related tubulointerstitial nephritis. Immunosuppressive therapy consisted of prednisone acetate (20 mg once daily) and mycophenolate mofetil (0.75 g twice daily).

Given the persistent electrolyte disturbances, aggressive electrolyte replacement was initiated, including potassium magnesium aspartate (0.28 g three times daily) and sustained-release potassium chloride (1 g three times daily).

In addition, the patient received concurrent management for hyperglycemia and liver dysfunction, including antidiabetic therapy and hepatoprotective treatment.

**Figure 2:**
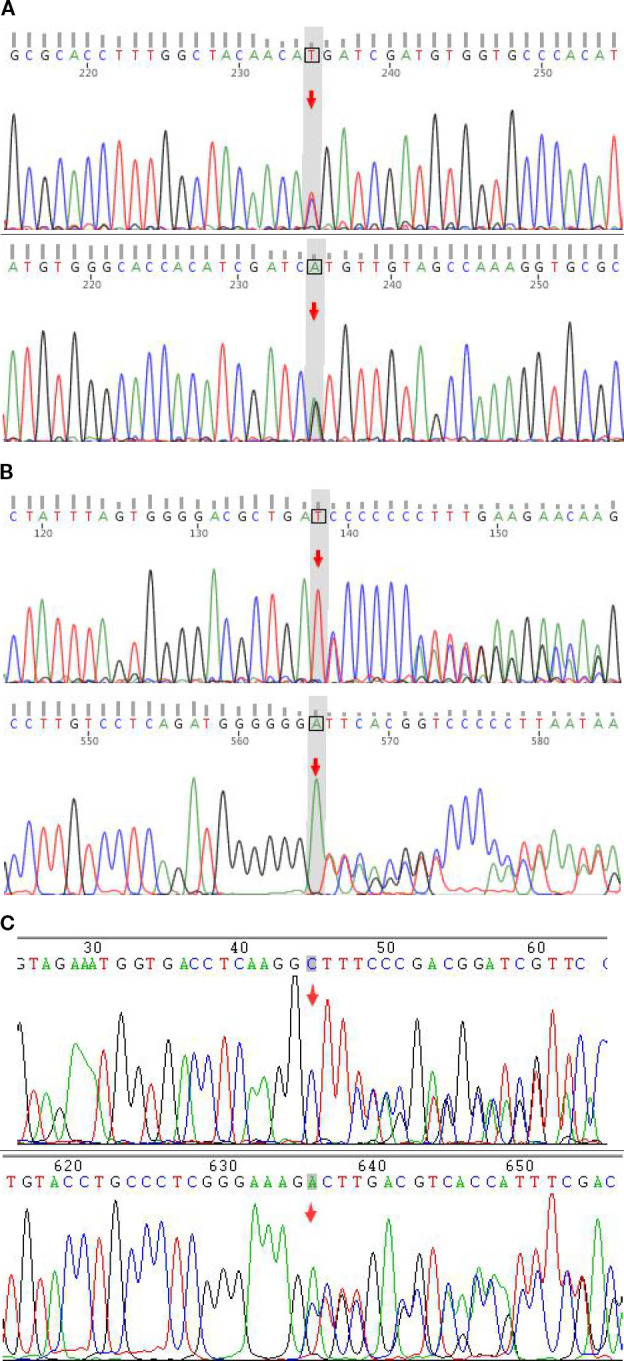
Representative forward and reverse sanger sequencing chromatograms of the patient. **(A)** SLC12A3 c.179C>T (p.Thr60Met), classified as pathogenic; **(B)** SLC12A3 c.917dup (p.Ser309Ilefs*2), classified as likely pathogenic;***(C)***MEFV c.712_725del (p.Met238Profs*2), classified as VUS.

Given the coexistence of tubulointerstitial nephritis-like manifestations with persistent hypokalemia, hypomagnesemia, and metabolic alkalosis confirmed by arterial blood gas analysis, inherited renal tubular disorders—including Gitelman syndrome and Fanconi syndrome—were considered in the differential diagnosis. Accordingly, genetic testing was pursued to further elucidate the underlying etiology. Genetic testing revealed 2 heterozygous mutations in the SLC12A3 gene: c.179C>T (p.Thr60Met) and c.917dup (p.Ser309Ilefs*2), confirming the diagnosis of Gitelman syndrome., and revealed 1 heterozygous mutations in the MEFV gene: c.712_725del(p.Met238Profs*2), as shown in **Figure 2**.

Based on the genetic findings and clinical reassessment, the diagnosis was refined to genetically confirmed GS with a suspected MEFV-associated autoinflammatory phenotype. The patient was treated with prednisone acetate (15 mg once daily) and colchicine (0.5 mg twice daily).For long-term management of electrolyte disturbances related to Gitelman syndrome, sustained-release potassium chloride (1 g three times daily) and spironolactone (20 mg twice daily) were administered in addition to ongoing magnesium supplementation. At follow-up evaluation, laboratory investigations demonstrated partial improvement of electrolyte abnormalities. Serum potassium remained mildly decreased at 3.09 mmol/L, and serum magnesium was 0.59 mmol/L. Serum sodium (139.6 mmol/L), chloride (99.8 mmol/L), calcium (2.53 mmol/L), and phosphorus (0.98 mmol/L) were within normal ranges. Inflammatory markers were within normal limits, with an erythrocyte sedimentation rate (ESR) of 21 mm/h and C-reactive protein (CRP) of 1.39 mg/L. Clinically, the patient reported no recurrent episodes of fever. Physical examination revealed no significant joint swelling or tenderness.

## 3 Discussion

We present a rare case of genetically confirmed Gitelman syndrome (GS) in a Chinese female patient who also exhibited systemic inflammatory manifestations and a heterozygous MEFV variant of uncertain significance. The combination of persistent renal tubular electrolyte disturbances and inflammatory features created substantial diagnostic complexity. Rather than representing two definitively established monogenic disorders, this case illustrates the challenges of interpreting genetic findings in patients with overlapping multisystem presentations. It underscores the importance of integrating molecular results with careful clinical evaluation to avoid overinterpretation of variants of uncertain significance in complex phenotypes.

The diagnosis of Gitelman syndrome (GS) was supported by persistent hypokalemia, hypomagnesemia, normotension, and metabolic alkalosis. Variant interpretation was performed in accordance with the standards and guidelines of the American College of Medical Genetics and Genomics (ACMG) and the Association for Molecular Pathology (AMP). The SLC12A3 c.179C>T (p.Thr60Met) variant was classified as pathogenic, and the c.917dup (p.Ser309Ilefs2) variant as likely pathogenic. In contrast, the MEFV c.712_725del (p.Met238Profs2) variant was classified as a variant of uncertain significance (VUS), as current evidence is insufficient to establish pathogenicity. These findings support a primary inherited renal tubular disorder as the explanation for the patient’s electrolyte abnormalities. Although the initial presentation with arthritis, elevated inflammatory markers, and positive labial gland biopsy raised suspicion for an autoimmune process, subsequent genetic analysis clarified that the renal tubular dysfunction was attributable to GS rather than secondary autoimmune involvement. This case underscores the importance of integrating clinical and molecular data when evaluating unexplained electrolyte disturbances, particularly in patients with overlapping inflammatory features.

Documented cases of coexisting familial Mediterranean fever (FMF) and Sjögren’s syndrome (SS) are rare but have been reported. In previously described cases, patients fulfilled established diagnostic criteria for both conditions and harbored pathogenic *MEFV* variants (6, 7). These observations suggest that overlap between autoinflammatory and autoimmune diseases, although uncommon, may occur in selected individuals. In the present case, focal lymphocytic infiltration on labial gland biopsy initially raised suspicion for SS. However, the patient lacked sicca symptoms, serological testing for anti-SSA/Ro, anti-SSB/La, and anti-Ro-52 antibodies was negative, and the total 2016 ACR/EULAR score was 3, below the diagnostic threshold. Therefore, primary SS was not established. The biopsy findings were interpreted cautiously as possibly reflecting nonspecific chronic inflammation rather than definitive autoimmune exocrinopathy. The patient also exhibited inflammatory arthritis and elevated acute-phase reactants. Genetic analysis identified a heterozygous *MEFV* frameshift variant (c.712_725del, p.Met238Profs*2), classified as a variant of uncertain significance according to ACMG criteria. Although certain *MEFV* variants have been associated with autosomal dominant FMF and atypical presentations (8), the current evidence is insufficient to establish a definitive diagnosis of FMF in this patient. Thus, the inflammatory manifestations are best described as a suspected autoinflammatory phenotype in the context of an *MEFV* variant. The absence of segregation analysis further limits causal inference.

This case highlights the challenges of interpreting *MEFV* variants in patients with atypical inflammatory presentations and underscores the need for cautious integration of genetic and clinical findings.

Although Gitelman syndrome (GS) is classically regarded as a monogenic renal tubular disorder, accumulating evidence indicates that it may coexist with autoimmune conditions, particularly autoimmune thyroid disease. Notably, the *SLC12A3* c.179C>T (p.Thr60Met) variant identified in our patient has been reported in cases of GS with autoimmune comorbidities (9). However, a causal relationship has not been established, and such overlap may reflect coincidence or shared immunological susceptibility.

GS with a suspected MEFV-associated inflammatory phenotype in this patient further highlights the diagnostic complexity that arises when inherited tubular dysfunction overlaps with systemic inflammatory features. Although it is conceivable that inflammatory activity could exacerbate tubular stress and destabilize electrolyte homeostasis in individuals with impaired NCC function, this hypothesis remains speculative and lacks direct mechanistic evidence. In our case, elevated inflammatory markers and focal lymphocytic infiltration on labial gland biopsy initially suggested an autoimmune etiology, yet comprehensive reassessment ultimately supported a primary genetic tubulopathy.

From a management perspective, treatment of GS remains supportive and follows consensus guidance, including recommendations from the Kidney Disease: Improving Global Outcomes (KDIGO) Controversies Conference. Long-term therapy centers on potassium and magnesium supplementation, monitoring of renal function and cardiac rhythm, and cautious adjustment of medications that may influence electrolyte balance. In patients with concomitant inflammatory disease, close multidisciplinary coordination is essential to balance electrolyte homeostasis with anti-inflammatory therapy.

## 4 Conclusion

This case describes a genetically confirmed Gitelman syndrome (GS) in a Chinese patient presenting with systemic inflammatory manifestations and a heterozygous MEFV variant of uncertain significance. Rather than representing two definitively established monogenic disorders, this case illustrates the diagnostic complexity that arises when inherited renal tubular dysfunction overlaps with inflammatory features.

Several important clinical principles emerge. First, precise diagnosis is essential. In patients with unexplained electrolyte disturbances accompanied by inflammatory findings, comprehensive biochemical evaluation and genetic testing are critical to distinguish primary inherited tubulopathies from secondary autoimmune or autoinflammatory conditions. Second, careful interpretation of genetic variants is necessary. The presence of an MEFV variant does not by itself confirm familial Mediterranean fever, particularly when established clinical criteria are not fulfilled. Third, multidisciplinary management is crucial to balance electrolyte replacement and control of inflammatory symptoms.

Accumulation of similar cases and further investigation will be important to clarify the clinical significance of MEFV variants in atypical inflammatory presentations and to refine diagnostic strategies for complex multisystem disorders.

## 5 Patient perspective

Written informed consent was obtained from the patient for publication of this case report and the accompanying clinical and genetic data.

## Data Availability

The datasets presented in this study can be found in online repositories. The names of the repository/repositories and accession number(s) can be found in the article/supplementary material.
